# Association of Albuminuria Within the Normoalbuminuric Range With All‐Cause Mortality in People With Type 2 Diabetes

**DOI:** 10.1002/dmrr.70061

**Published:** 2025-06-25

**Authors:** Monia Garofolo, Giuseppe Penno, Anna Solini, Emanuela Orsi, Martina Vitale, Veronica Resi, Enzo Bonora, Cecilia Fondelli, Roberto Trevisan, Monica Vedovato, Antonio Nicolucci, Giuseppe Pugliese, Renal Insufficiency And Cariovascular Events (RIACE) Study Group

**Affiliations:** ^1^ Department of Clinical and Experimental Medicine University of Pisa Pisa Italy; ^2^ Department of Surgical, Medical, Molecular and Critical Area Pathology University of Pisa Pisa Italy; ^3^ Diabetes Unit Fondazione IRCCS “Cà Granda ‐ Ospedale Maggiore Policlinico” Milan Italy; ^4^ Department of Clinical and Molecular Medicine “La Sapienza” University Rome Italy; ^5^ Division of Endocrinology Diabetes and Metabolism University and Hospital Trust of Verona Verona Italy; ^6^ Diabetes Unit University of Siena Siena Italy; ^7^ Endocrinology and Diabetes Unit Azienda Ospedaliera Papa Giovanni XXIII Bergamo Italy; ^8^ Department of Clinical and Experimental Medicine University of Padua Padua Italy; ^9^ Centre for Outcomes Research and Clinical Epidemiology (CORESEARCH) Pescara Italy

**Keywords:** albuminuria, all‐cause mortality, chronic kidney disease, estimated glomerular filtration rate, type 2 diabetes

## Abstract

**Aims:**

To investigate the independent association of albuminuria within the normoalbuminuric range with all‐cause mortality in normoalbuminuric people with type 2 diabetes with and without chronic kidney disease (CKD).

**Materials and Methods:**

This observational, prospective, multicentre, cohort study enroled 15,773 individuals with type 2 diabetes in 2006–2008. At baseline, albumin excretion rate (AER) and estimated glomerular filtration rate (eGFR) were assessed together with cardiometabolic risk profile, treatments, complications, and comorbidities. All‐cause mortality was verified on 31 October 2015.

**Results:**

Of the 15,656 participants (99.3%) with valid information on vital status, 11,460 (71.2%) were normoalbuminuric, 9984 (87.1%) without and 1476 (12.9%) with CKD. Normoalbuminuric individuals were stratified into three (< 5, 5–15, and > 15 mg·day^−1^) or two (< 10 and 10–29 mg·day^−1^) AER subcategories. When adjusting for age, sex, eGFR, prior cardiovascular disease, cardiovascular risk factors, and treatments, mortality risk was higher in participants with AER 10–29 versus < 10 mg·day^−1^ (hazard ratio, 1.120 [95% confidence interval, 1.028–1.221], *p* = 0.009) and 15–29 versus < 5 mg·day^−1^ (1.243 [1.099–1.406], *p* < 0.0001). When stratifying by CKD status, the adjusted risk remained significantly increased only for AER 15–29 versus  < 5 mg/24 h in individuals with (1.404 [1.111–1.774], *p* = 0.005) and, to a lesser extent, without (1.167 [1.009–1.350], *p* = 0.038) CKD. A non‐linear association was observed between AER as Log2 transformed continuous variable and mortality.

**Conclusions:**

For the same level of kidney function, higher AER within the normoalbuminuric range was independently associated with all‐cause mortality, thus supporting to the use of albuminuria‐lowering drugs in people with type 2 diabetes and mildly elevated albuminuria.

**Trial Registration:**

ClinicalTrials.gov, NCT00715481, retrospectively registered 15 July, 2008.

## 1 Introduction

In the last decades, nonalbuminuric renal impairment has become the prevailing chronic kidney disease (CKD) phenotype in people with diabetes, especially type 2, and reduced estimated glomerular filtration rate (eGFR) (i.e., < 60 mL·min^−1^·1.73 m^−2^) [1], pointing to the existence of a nonproteinuric pathway to loss of kidney function in these individuals [2]. Several studies have shown that the nonalbuminuric CKD phenotype is associated with an increased risk of all‐cause death and morbidity and mortality from cardiovascular disease (CVD), which is similar to that of albuminuric CKD with preserved eGFR (i.e., micro or macroalbuminuria and eGFR ≥ 60 mL·min^−1^·1.73 m^−2^) and lower than that of albuminuric CKD with reduced eGFR (i.e., micro or macroalbuminuria and eGFR < 60 mL·min^−1^·1.73 m^−2^) [3, 4, 5, 6]. In particular, among participants in the Renal Insufficiency And Cardiovascular Events (RIACE) Italian Multicenter Study, nonalbuminuric individuals with an eGFR in the G3a category (45–59 mL·min^−1^·1.73 m^−2^) had the same adjusted risk of death as those with microalbuminuria alone, whereas nonalbuminuric individuals with an eGFR in the G3b category (30–44 mL·min^−1^·1.73 m^−2^) had the same adjusted risk of death as those with macroalbuminuria alone [4]. Conversely, a post hoc analysis of the Action in Diabetes and Vascular disease: preterAx and diamicroN‐MR Controlled Evaluation (ADVANCE) showed that progression to end‐stage kidney disease (ESKD) was lower in people with nonalbuminuric CKD compared with those with the albuminuric phenotypes [3]. A lower or even no risk of progression to ESKD was also shown in participants in the Hong Kong Diabetes Biobank Study [5] and in a post hoc analysis of the Action to Control Cardiovascular Risk in Diabetes (ACCORD) Study [6], respectively, thus supporting the concept that albuminuria is a major risk factor for CKD progression [7]. Moreover, an analysis of individuals with either type 1 or type 2 diabetes and reduced eGFR attending the Steno Diabetes Center showed that eGFR declined also in those with normoalbuminuria, with a rate that was only slightly less than in those with microalbuminuria (1.9 vs. 2.1 mL·min^−1^·1.73 m^−2^ per year) but much less than in those with macroalbuminuria [8].

In addition to the impact of reduced eGFR per se, several studies have shown that levels of albuminuria within the normoalbuminuric range are independently associated with adverse cardiorenal outcomes in both the general population and high‐risk individuals, with risk increasing at an albumin:creatinine ratio (ACR) of ∼10 mg·g^−1^ [9, 10, 11, 12, 13]. This prompted the identification of two subcategories within the normoalbuminuria category, that is, normal (ACR < 10 mg·g^−1^) and mildly increased (ACR 10–29 mg·g^−1^) [14]. However, data were mainly derived from people with relatively preserved kidney function, thus not allowing to separate the impact of higher albuminuria within the normoalbuminuric range from that of reduced eGFR. Recently, Verma et al. have reported that, in CKD participants in the Chronic Renal Insufficiency Cohort (CRIC) Study, risk for CKD progression increased within the normoalbuminuric range and was significantly higher in participants with ACR > 15 mg·g^−1^ than in those with ACR < 5 mg·g^−1^ [15].

This analysis aimed to assess the independent association of albuminuria within the normoalbuminuric range with all‐cause mortality in normoalbuminuric individuals with type 2 diabetes from the RIACE cohort, either as a whole or separately in participants with and without CKD.

## 2 Materials and Methods

### 2.1 Design and Participants

The RIACE Italian Multicenter Study was an observational, prospective, multicentre, cohort study on the impact of eGFR on morbidity and mortality in people with type 2 diabetes [16].

A total of 15,773 Caucasian individuals with type 2 diabetes consecutively attending 19 hospital‐based, tertiary referral Diabetes Clinics of the National Health Service throughout Italy, were enroled in the years 2006–2008, after excluding 160 individuals with missing or implausible values. Exclusion criteria were dialysis or kidney transplantation.

### 2.2 Baseline Data

Baseline data were collected using a standardized protocol across participating centres; results from different laboratories/methods were standardized by comparison with values detected in test samples at the reference laboratory of the Coordinating Centre [16].

Participants underwent a structured interview to collect the following information: current age, smoking status, known diabetes duration, severe co‐morbidities, and current treatments including glucose‐, lipid‐, and blood pressure (BP)‐lowering therapies.

Body mass index (BMI) was calculated from weight and height, whereas estimated waist circumference (eWC) was calculated from Log‐transformed BMI values [17]. Then, BP was measured using a sphygmomanometer with the patients seated with the arm at the heart level.

Haemoglobin A_1c_ (HbA_1c_) was measured by HPLC using DCCT‐aligned methods, whereas triglycerides and total and HDL cholesterol were determined in fasting blood samples by standard colourimetric enzymatic methods. Then, the LDL cholesterol concentration was estimated using the Friedewald formula.

The presence of CKD was assessed by measuring albuminuria and serum creatinine, as previously detailed [4, 16]. Briefly, albumin excretion rate (AER) was obtained from 24‐h urine collections or estimated from ACR in early‐morning first‐voided urine samples using a conversion formula developed in people with type 1 diabetes [18], in keeping with a report showing that estimated AER is more accurate than measured ACR in predicting measured AER [19]. Using estimated AER instead of measured ACR led to the reclassification of only 129 participants (0.82%), with 116 individuals changing from micro to normoalbuminuria or vice versa (100 and 16, respectively) [4]. Albumin concentration in urine was measured by immunonephelometry or immunoturbidimetry in the absence of interfering clinical conditions. One‐to‐three measurements for each participant were obtained; in cases of multiple measurements, the geometric mean of 2–3 values was used for analysis. In individuals with multiple measurements, the concordance rate between the first value and the geometric mean was > 90% for all albuminuria categories [20]. Serum (and urine) creatinine was measured by the modified Jaffe method, traceable to IDMS, and GFR was estimated using the 2009 CKD epidemiology collaboration (CKD‐EPI) equation [21].

The presence of diabetic retinopathy (DR) was assessed in each centre by an expert ophthalmologist by dilated fundoscopy [22]. Participants were then classified as having no DR, non‐advanced DR (including mild or moderate non‐proliferative DR), or advanced DR (including severe non‐proliferative DR, proliferative DR, or diabetic macular oedema). The DR grade was assigned based on the worse eye.

Previous major adverse CVD events, including myocardial infarction, stroke, foot ulcer, gangrene and non‐traumatic amputation, and cerebrovascular, carotid, and lower limb revascularisation, were adjudicated based on hospital discharge records by an ad hoc committee in each centre [23].

### 2.3 All‐Cause Mortality

The vital status of study participants on 31 October 2015 was verified by interrogating the Italian Health Card database (http://sistemats1.sanita.finanze.it/wps/portal/), which provides updated and reliable information on all current Italian residents [24].

### 2.4 Statistical Analysis

Data are expressed as mean ± SD or median (interquartile range, IQR) for continuous variables, and number of cases (percentage) for categorical variables. The Kolmogorov‐Smirnov test was used to determine if variables were normally distributed. Continuous variables were compared using the Student's *t*‐test (or one‐way ANOVA) and Mann‐Whitney test (or Kruskal‐Wallis's test) for parametric and non‐parametric data, respectively, whereas the χ^2^ test was applied to categorical variables. None of the variables had missing values.

Participants with normoalbuminuria were considered for this analysis and stratified either in three AER subcategories (< 5, 5–15, and > 15 mg·day^−1^)as in Verma et al. [15], or in two AER subcategories (< 10 and 10–29 mg·day^−1^), according to KDIGO [14].

Crude mortality rates according to AER subcategories were described as events per 1000 patient‐years from start of follow‐up to censoring, with 95% exact Poisson confidence intervals (CIs) and adjusted for age and sex by a Poisson regression model. Kaplan‐Meier survival curves for all‐cause mortality were calculated according to AER subcategories and differences were analysed with the log‐rank statistic. In addition, Cox proportional hazards regression analyses with backward variable selection were run according to AER subcategories or AER as a continuous variable, either unadjusted or sequentially adjusted for age and sex (model 1), eGFR and prior CVD (model 2), and smoking, diabetes duration, HbA_1c_, BMI, dyslipidemia, hypertension, and use of renin‐angiotensin system (RAS) blockers (model 3). These analyses were performed in the whole cohort and separately in participants with and without CKD (i.e., with an eGFR < 60 and ≥ 60 mL/min/1.73 m^2^, respectively). As AER was not normally distributed, data were Log2 transformed before use as a continuous variable. In addition, the multiplicative interaction term (AER *x* CKD Yes/no) was included as a covariate in the Cox regression models to assess whether CKD status modified the association between AER (AER subcategories or AER as a continuous variable) and all‐cause mortality. Finally, to assess whether AER (as Log2 transformed values) was non‐linearly associated with all‐cause mortality, Cox proportional hazard regressions were run using restricted cubic splines.

Tests were two sided, and a *p* value < 0.05 was considered statistically significant. Data entry and statistical analyses were performed using SPSS version 26.0 (SPSS, Chicago, IL, USA) and R statistical software version 4.0.4 (https://cran.r‐project.org/index.html).

## 3 Results

Of the 15,656 participants (99.3% of the cohort) with valid information on vital status, 11,460 (718.2%) were normoalbuminuric. They had a mean age of 66.0 ± 10.3 years, 47.1% were females, median AER was 9.43 (5.14–15.50) mg·day^−1^ and mean eGFR was 82.7 ± 18.9 mL·min^−1^·1.73 m^−2^. Of these individuals, 9984 (87.1%) had preserved eGFR (87.7 ± 14.1 mL·min^−1^·1.73 m^−2^, no CKD) and 1476 (12.9%) had reduced eGFR (48.5 ± 9.5 mL·min^−1^·1.73 m^−2^, CKD). Compared with individuals without CKD, those with CKD were older (74.4 ± 8.1 vs. 64.8 ± 9.9 years, *p* < 0.0001) and less frequently female (39.8 vs. 45.2%, *p* < 0.0001) and had slightly higher AER levels (10.52 [5.65–17.28] vs. 9.34 [5.14–15.20] mg·day^−1^, *p* < 0.0001).

The baseline clinical features of normoalbuminuric participants stratified by three and two AER subgroups are shown in Table 1 and Supporting Information S1: Table S1, respectively. As a general trend, the cardiometabolic risk profile worsened, whereas age, diabetes duration, and prevalence of male sex, smoking, treatments, complications, and comorbidities increased from the lowest to the highest AER subcategories.

**TABLE 1:** Baseline clinical features in all normoalbuminuric participants and those without and with CKD, stratified by three AER subcategories (< 5, 5–14, and 15–29 mg·day^−1^).

	All (*n*. 11,460)				Without CKD (*n* = 9984)				With CKD (*n* = 1476)			
	< 5	5–14	15–29	*p*	< 5	5–14	15–29	*p*	< 5	5–14	15–29	*p*
*N* (%)	2606 (22.7)	5746 (50.1)	3108 (27.1)		2313 (23.2)	5048 (50.5)	2623 (26.3)		293 (19.9)	698 (47.2)	485 (32.9)	
AER, mg·day^−1^	3.00 (1.44–3.96)	9.05 (6.91–11.6)	19.7 (17.1–23.7)		3.00 (1.44–3.96)	9.03 (6.91–11.52)	19.6 (17.0–23.6)		2.86 (1.38–3.92)	9.34 (6.78–11.90)	20.0 (17.3–24.3)	
Age, years	65.0 ± 10.1	65.9 ± 10.3	67.1 ± 10.2	< 0.0001	63.9 ± 9.9	64.7 ± 10.0	65.7 ± 9.9	< 0.0001	73.6 ± 7.6	74.5 ± 7.8	74.7 ± 8.8	0.162
Sex, *n* (%)				< 0.0001				< 0.0001				< 0.0001
Females	1315 (50.5)	2815 (49.0)	1269 (40.8)		1124 (48.6)	2372 (47.0)	1014 (38.7)		191 (65.2)	443 (63.5)	255 (52.6)	
Males	1291 (49.5)	2931 (51.0)	1839 (59.2)		1189 (51.4)	2676 (53.0)	1609 (61.3)		102 (34.8)	255 (36.5)	230 (47.4)	
Smoking, *n* (%)				< 0.0001				< 0.0001				0.009
Never	1689 (64.8)	3306 (57.5)	1.750 (56.3)		1480 (64.0)	2878 (57.0)	1452 (55.4)		209 (71.3)	428 (61.3)	298 (61.4)	
Former	590 (22.6)	1578 (27.5)	893 (28.7)		532 (23.0)	1363 (27.0)	749 (28.6)		58 (19.8)	215 (30.8)	144 (29.7)	
Current	327 (12.5)	862 (15.0)	465 (15.0)		301 (13.0)	807 (16.0)	422 (16.1)		26 (8.9)	55 (7.9)	43 (8.9)	
Diabetes duration, years	11.6 ± 9.6	12.5 ± 10.0	13.2 ± 10.2	< 0.0001	11.0 ± 9.2	12.0 ± 9.7	12.5 ± 9.8	< 0.0001	15.8 ± 11.1	16.2 ± 11.2	17.1 ± 11.4	0.252
HbA_1c_, %	7.30 ± 1.31	7.44 ± 1.43	7.51 ± 1.46	< 0.0001	7.27 ± 1.30	7.42 ± 1.44	7.49 ± 1.45	< 0.0001	7.49 ± 1.43	7.59 ± 1.41	7.63 ± 1.51	0.399
(mmol·mol^−1^)	(56.3 ± 14.4)	(57.8 ± 15.7)	(58.6 ± 16.0)	< 0.0001	(56.0 ± 14.2)	(57.6 ± 15.7)	(58.3 ± 15.9)	< 0.0001	(58.3 ± 15.6)	(59.4 ± 15.4)	(59.9 ± 16.5)	0.399
BMI, kg·m^−2^	28.6 ± 5.1	28.7 ± 5.0	29.0 ± 5.2	0.014	28.6 ± 5.1	28.7 ± 5.0	29.0 ± 5.2	0.003	29.1 ± 5.0	29.1 ± 5.2	28.9 ± 5.0	0.665
eWC, cm	101.6 ± 10.2	101.9 ± 10.1	102.6 ± 10.6	< 0.0001	101.5 ± 10.3	101.8 ± 10.1	102.7 ± 10.7	< 0.0001	102.3 ± 9.8	102.4 ± 10.3	102.1 ± 10.1	0.881
Triglycerides, mmol·l^−1^	1.28 (0.94–1.74)	1.27 (0.93–1.80)	1.34 (0.97–1.90)	< 0.0001	1.27 (0.93–1.70)	1.25 (0.91–1.74)	1.32 (0.95–1.88)	< 0.0001	1.43 (1.09–2.11)	1.43 (1.08–2.03)	1.50 (1.04–2.04)	0.867
Total cholesterol, mmol·l^−1^	4.77 ± 0.94	4.79 ± 0.97	4.78 ± 0.98	0.477	4.75 ± 0.93	4.78 ± 0.97	4.78 ± 0.97	0.147	4.91 ± 1.02	4.76 ± 1.00	4.72 ± 1.04	0.040
HDL cholesterol, mmol·l^−1^	1.27 (1.06–1.53)	1.27 (1.07–1.50)	1.24 (1.03–1.47)	< 0.0001	1.29 (1.07–1.53)	1.27 (1.09–1.52)	1.26 (1.03–1.50)	0.001	1.24 (1.06–1.45)	1.21 (1.03–1.47)	1.16 (0.98–1.42)	0.021
Non‐HDL cholesterol, mmol·l^−1^	3,44 ± 0.89	3.48 ± 0.94	3.49 ± 0.95	0.170	3.42 ± 0.88	3.48 ± 0.94	3.49 ± 0.94	0.021	3.63 ± 0.97	3.49 ± 0.93	3.49 ± 0.97	0.075
LDL cholesterol, mmol·l^−1^	2.78 ± 0.82	2.81 ± 0.84	2.79 ± 0.84	0.143	2.78 ± 0.81	2.82 ± 0.84	2.79 ± 0.84	0.025	2.86 ± 0.86	2.74 ± 0.85	2.75 ± 0.88	0.115
Dyslipidaemia, *n* (%)	2114 (81.1)	4761 (82.9)	2556 (82.2)	0.155	1860 (80.4)	4177 (82.7)	2153 (82.1)	0.054	254 (86.7)	484 (83.7)	403 (83.1)	0.381
Systolic BP, mmHg	135.9 ± 17.7	137.2 ± 17.5	138.1 ± 17.4	< 0.0001	136.0 ± 17.6	137.0 ± 17.3	138.0 ± 17.2	< 0.0001	138.1 ± 18.0	138.6 ± 19.4	138.9 ± 18.2	0.838
Diastolic BP, mmHg	78.2 ± 9.5	78.7 ± 9.1	79.0 ± 9.2	0.003	78.3 ± 9.4	78.8 ± 9.0	79.3 ± 9.1	< 0.0001	77.4 ± 9.8	77.5 ± 9.7	77.3 ± 9.7	0.927
Mean BP, mmHg	97.4 ± 10.5	98.2 ± 10.2	98.7 ± 10.3	< 0.0001	97.4 ± 10.5	98.2 ± 10.1	98.9 ± 10.3	< 0.0001	97.7 ± 10.4	97.9 ± 11.0	97.8 ± 10.8	0.967
Pulse pressure, mmHg	57.7 ± 15.5	58.6 ± 15.4	59.1 ± 15.1	0.004	57.4 ± 15.2	58.2 ± 15.1	58.6 ± 14.8	0.012	60.7 ± 16.8	61.1 ± 17.4	61.7 ± 16.0	0.711
Hypertension, *n* (%)	2035(78.1)	4665 (81.2)	2577 (82.9)	< 0.0001	1765 (76.3)	4009 (79.4)	2132 (81.3)	< 0.0001	270 (92.2)	656 (94.0)	445 (91.8)	0.293
Anti‐hyperglycaemic Tx, *n* (%)				< 0.0001				< 0.0001				0.002
Lifestyle only	466 (17.3)	906 (15.8)	366 (11.8)		425 (18.4)	820 (16.2)	330 (12.6)		41 (14.0)	86 (12.3)	36 (7.4)	
Non‐insulin	1586(60.9)	3675 (64.0)	1984 (63.8)		1429 (61.8)	3259 (64.6)	1713 (65.3)		157 (53.6)	416 (59.6)	271 (55.9)	
Insulin	554 (21.3)	1165 (20.3)	758 (24.4)		459 (19.8)	969 (19.2)	580 (22.1)		95 (32.4)	196 (28.1)	178 (36.7)	
Lipid‐lowering Tx, *n* (%)	1169 (44.9)	2617 (45.5)	1403 (45.1)	0.830	1003 (43.4)	2233 (44.2)	1146 (43.7)	0.761	166 (56.7)	384 (55.0)	257 (53.0)	0.591
Anti‐hypertensive Tx, *n* (%)	1643 (63.0)	3827 (66.6)	2148 (69.1)	< 0.0001	1393 (60.2)	3224 (63.9)	1731 (66.0)	< 0.0001	250 (85.3)	603 (86.4)	417 (86.0)	0.906
RAS blockers, *n* (%)	1295 (49.7)	3096 (53.9)	1747 (56.2)	< 0.0001	1096 (47.4)	2613 (51.8)	1401 (53.4)	< 0.0001	199 (67.9)	483 (69.2)	346 (71.3)	0.566
Anti‐platelet Tx, *n* (%)	870 (33.4)	2123 (36.9)	1217 (39.2)	< 0.0001	715 (30.9)	1760 (34.9)	953 (36.3)	< 0.0001	155 (52.9)	363 (52.0)	264 (54.4)	0.713
Anti‐coagulant Tx, *n* (%)	71 (2.7)	178 (3.1)	140 (4.5)	< 0.0001	50 (2.2)	126 (2.5)	88 (3.4)	0.022	21 (7.2)	52 (7.4)	52 (10.7)	0.093
Serum creatinine, μmol/L	79.9 ± 20.6	78.7 ± 20.3	83.0 ± 27.5	< 0.0001	75.5 ± 14.6	74.1 ± 14.5	75.8 ± 14.8	< 0.0001	114.2 ± 27.3	111.9 ± 24.9	121.6 ± 43.6	< 0.0001
eGFR, ml·min^−1^·1.73 m^−2^	83.2 ± 18.4	83.3 ± 18.4	81.2 ± 20.2	< 0.0001	87.5 ± 14.1	88.0 ± 13.9	87.5 ± 14.5	0.237	48.7 ± 9.1	49.2 ± 8.9	47.2 ± 10.4	0.002
DR, *n* (%)				< 0.0001				< 0.0001				0.001
No	2214 (85.0)	4725 (82.2)	2463 (79.2)		1985 (85.8)	4184 (82.9)	2.104 (80.2)		229 (78.2)	541 (77.5)	359 (74.0)	
Non‐advanced	209 (8.0)	662 (11.5)	389 (12.5)		185 (8.0)	557 (11.0)	232 (12.3)		24 (8.2)	105 (15.0)	66 (13.5)	
Advanced	183 (7.0)	359 (6.2)	256 (8.2)		143 (6.2)	107 (6.1)	196 (7.5)		40 (13.7)	52 (7.4)	60 (12.4)	
CVD, *n* (%)												
Any	435 (16.7)	1133 (19.7)	697 (22,4)	< 0.0001	340 (14.7)	901 (17.8)	529 (20.2)	< 0.0001	95 (32.4)	232 (33.2)	168 (34.6)	0.796
Acute myocardial infarction	240 (9.2)	550 (9.6)	348 (11.2)	0.019	190 (8.2)	433 (8.6)	257 (9.8)	0.103	50 (17.1)	117 (16.8)	91 (18.8)	0.658
Coronary revascularisation	202 (7.8)	525 (9.1)	312 (10.0)	0.011	157 (6.8)	416 (8.2)	236 (9.0)	0.016	45 (15.4)	109 (15.6)	76 (15.7)	0.993
Any coronary event	313 (12.0)	778 (13.5)	478 (15.4)	0.001	245 (10.6)	615 (12.2)	358 (13.6)	0.005	68 (23.2)	163 (23.4)	120 (24.7)	0.831
Stroke	68 (2.6)	130 (2.3)	103 (3.3)	0.013	55 (2.4)	94 (1.9)	82 (3.1)	0.002	13 (4.4)	36 (5.2)	21 (4.3)	0.775
Carotid revascularisation	80 (3.1)	281 (4.9)	138 (4.4)	0.001	57 (2.5)	215 (4.3)	98 (3.7)	0.001	23 (7.8)	66 (9.5)	40 (8.2)	0.642
Any cerebrovascular event	142 (5.4)	392 (6.8)	226 (7.3)	0.016	108 (4.7)	297 (5.9)	168 (6.4)	0.027	34 (11.6)	95 (13.6)	58 (12.0)	0.583
Ulcer/gangrene/amputation	27 (1.0)	133 (2.3)	91 (2.9)	< 0.0001	18 (0.8)	102 (2.0)	64 (2.4)	< 0.0001	9 (3.1)	31 (4.4)	27 (5.6)	0.265
Lower limb revascularisation	49 (1.9)	123 (2,1)	74 (2.4)	0.429	37 (1.6)	90 (1.8)	51 (1.9)	0.659	12 (4.1)	33 (4.7)	23 (4.7)	0.897
Any peripheral event	69 (2.6)	247 (4.3)	153 (4.9)	< 0.0001	50 (2.2)	186 (3.7)	110 (4.2)	< 0.0001	19 (6.5)	61 (8.7)	43 (8.9)	0.440
Comorbidities, *n* (%)												
Any	412 (15.8)	936 (16.3)	561 (18.1)	0.044	357 (15.4)	806 (16.0)	447 (17.0)	0.281	55 (18.8)	130 (18.6)	114 (23.5)	0.094
COPD	88 (3.4)	205 (3.6)	135 (4.3)	0.101	71 (3.1)	166 (3.3)	94 (3.6)	0.596	17 (5.8)	39 (5.6)	41 (8.5)	0.124
Chronic liver disease	206 (7.9)	472 (8.2)	270 (8.7)	0.550	179 (7.7)	415 (8.2)	231 (8.8)	0.392	27 (9.2)	57 (8.2)	39 (8,0)	0.828
Cancer	153 (5.9)	337 (5.9)	220 (7.1)	0.057	136 (5.9)	291 (5.8)	171 (6.5)	0.405	17 (5.8)	46 (6.6)	49 (10.1)	0.035

*Note:* Data are expressed as mean ± SD or median (interquartile range), for continuous variables, and number of cases (percentage), for categorical variables.

Abbreviations: AER = albumin excretion rate, BMI = body mass index, BP = blood pressure, CKD = chronic kidney disease, COPD = chronic obstructive pulmonary disease, CVD = cardiovascular disease, DR = diabetic retinopathy, eGFR = estimated glomerular filtration rate, eWC = estimated waist circumference, HbA_1c_ = haemoglobin A_1c_, Tx = treatment.

After a median follow‐up of 8.08 (7.61–8.54) years, of the 11,460 participants with normoalbuminuria, 9320 (81.3%) were alive, whereas 2140 (18.7%) were deceased at the time of the census. The AER levels were slightly but significantly higher in those who died than in those who survived; this was the case when considering all normoalbuminuric individuals (10.82 [6.04–17.28] vs. 9.21 [5.03–15.00] mg·day^−1^, *p* < 0.0001) as well as those without (10.44 [6.00–16.55] vs. 9.10 [5.00–15.00] mg·day^−1^, *p* < 0.0001) and with (11.83 [6.19–18.78] vs. 9.91 [5.14–16.20] mg·day^−1^, *p* = 0.002) CKD separately.

**FIGURE 1:**
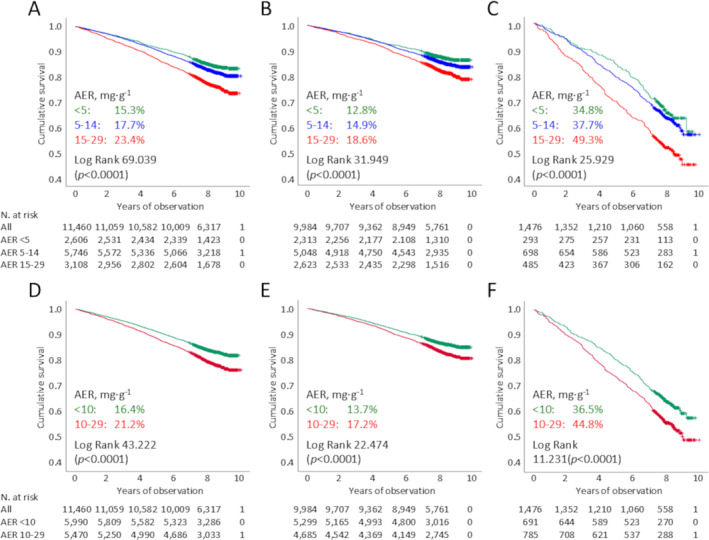
Kaplan Meier analysis for all‐cause mortality in normoalbuminuric individuals stratified in three (< 5, 5–15, and > 15 mg·day^−1^, Panels (A–C)) or two (< 10 and 10–29 mg·day^−1^, Panels (D–F)) AER subcategories in the whole group (Panels A, D) and in participants without (Panels B, E) and with (Panels C, F) CKD. Numbers (percentages) of deaths, log rank (*p* value) and number at risk are shown for each AER subcategory. AER = albumin excretion rate; CKD = chronic kidney disease.

As expected, death percentages and rates (Table 2) were much higher in normoalbuminuric participants with versus without CKD (*p* < 0.0001). In addition, death percentages and rates (Table 2), Kaplan‐Meier estimates (Figure 1), and unadjusted HRs (Table 3) increased in a stepwise manner across AER subcategories in the whole normoalbuminuric group and in individuals without and with CKD. When adjusted for all confounders, mortality risk was higher in normoalbuminuric participants with AER 10–29 versus. < 10 mg·day^−1^ and 15–29 versus. < 5 mg·day^−1^; however, when they were stratified according to CKD status, the adjusted risk of death remained significantly increased only for AER 15–29 versus. < 5 mg·day^−1^ in individuals with and, to a lesser extent, without CKD (Table 3). An independent association with mortality was also observed when AER was included as a Log2 transformed continuous variable (Table 4). The global *p*‐value for non‐linearity was < 0.001, pointing to a non‐linear relationship between AER within the normoalbuminuric range and mortality, as shown in Supporting Information S1: Figure 1.

**TABLE 2:** Mortality rates in all normoalbuminuric participants and in those without and with CKD, stratified by three (< 5, 5–14, and 15–29 mg·day^−1^) or two (< 10 and 10–29 mg·day^−1^) AER subcategories.

	*N*	Events	Percent events	Events per 1000 patient‐years (95% CI), unadjusted	*p*	Events per 1000 patient‐years (95% CI), age‐ & sex‐adjusted	*p*
All	11,460	2140	18.7	24.6 (23.5–25.6)		10.6 (9.1–12.3)	
< 5 mg·day^−1^	2606	398	15.3	19.8 (17.9–21.8)	Ref	9.8 (8.3–11.6)	Ref
5–14 mg·day^−1^	5746	1016	17.7	23.1 (21.7–24.6)	0.007	10.4 (8.9–12.1)	0.342
15–29 mg·day^−1^	3108	726	23.4	31.5 (29.3–33.9)	< 0.0001	12.7 (10.8–15.1)	< 0.0001
< 10 mg·day^−1^	5990	980	16.4	21.3 (20.0–22.7)	Ref	10.0 (8.6–11.7)	Ref
10–29 mg·day^−1^	5470	1160	21.2	28.2 (26.6–29.9)	< 0.0001	11.6 (9.9–13.6)	< 0.0001
Without CKD	9984	1536	15.4	19.9 (18.9–20.9)		8.5 (7.1–10.2)	
< 5 mg·day^−1^	2313	296	12.8	16.4 (14.6–18.4)	Ref	7.9 (6.4–9.7)	Ref
5–14 mg·day^−1^	5048	753	14.9	19.2 (17.9–20.6)	0.017	8.5 (7.1–10.2)	0.275
15–29 mg·day^−1^	2623	487	18.6	24.3 (22.2–26.6)	< 0.0001	9.7 (8.0–11.9)	0.005
< 10 mg·day^−1^	5299	728	13.7	17.6 (16.4–19.0)	Ref	8.1 (6.8–9.8)	Ref
10–29 mg·day^−1^	4685	808	17.2	22.4 (20.9–24.0)	< 0.0001	9.2 (7.6–11.1)	0.020
With CKD	1476	604	40.9	61.5 (56.8–66.6)		20.6 (15.4–27.5)	
< 5 mg·day‐1	293	102	34.8	49.8 (41.0–60.4)	Ref	19.1 (13.7–26.4)	Ref
5–14 mg·day‐1	698	263	37.7	55.1 (48.8–62.2)	0.374	19.7 (14.6–26.6)	0.788
15–29 mg·day‐1	485	239	49.3	79.7 (70.2–90.5)	< 0.0001	27.7 (20.1–40.0)	< 0.0001
< 10 mg·day‐1	691	252	36.5	53.1 (47.0–60.1)	Ref	19.1 (14.2–1.74)	Ref
10–29 mg·day‐1	785	352	44.8	69.3 (62.4–76.9)	0.001	23.3 (17.2–31.6)	0.017

Abbreviations: AER = albumin excretion rate, CI = confidence interval, CKD = chronic kidney disease.

**TABLE 3:** Cox proportional hazards regression with backward selection of variables for all‐cause mortality in all normoalbuminuric participants and in those without and with CKD according to three (< 5, 5–14, and 15–29 mg·day^−1^) or two (< 10 and 10–29 mg·day^−1^) AER subcategories.

	Unadjusted		Model 1		Model 2		Model 3	
	HR (95% CI)	*p*	HR (95% CI)	*p*	HR (95% CI)	*p*	HR (95% CI)	*p*
All		< 0.0001		< 0.0001		< 0.0001		< 0.0001
< 5 mg·day^−1^	1	—	1	—	1	—	1	—
5–14 mg·day^−1^	1.168 (1.040–1.311)	0.009	1.059 (0.943–1.189)	0.332	1.060 (0.944–1.191)	0.325	1.031 (0.918–1.159)	0.602
15–29 mg·day^−1^	1.598 (1.414–1.806)	< 0.0001	1.311 (1.159–1.482)	< 0.0001	1.287 (1.138–1.456)	< 0.0001	1.243 (1.099–1.406)	< 0.0001
*p* AER × CKD		0.389		0.168		0.179		0.178
< 10 mg·day^−1^	1		1		1		1	
10–29 mg·day^−1^	1.329 (1.220–1.447)	< 0.0001	1.165 (1.070–1.269)	< 0.0001	1.146 (1.052–1.248)	0.002	1.120 (1.028–1.221)	0.009
*p* AER × CKD		0.733		0.520		0.534		0.608
Without CKD		< 0.0001		< 0.0001		< 0.0001		0.046
< 5 mg·day^−1^	1		1		1		1	
5–14 mg·day^−1^	1.171 (1.024–1.340)	0.021	1.076 (0.940–1.231)	0.289	1.054 (0.921–1.206)	0.448	1.027 (0.897–1.176)	0.700
15–29 mg·day^−1^	1.485 (1.285–1.715)	< 0.0001	1.234 (1.067–1.427)	0.005	1.205 (1.042–1.394)	0.012	1.167 (1.009–1.350)	0.038
< 10 mg·day^−1^	1		1		1		1	
10–29 mg·day^−1^	1.273 (1.152–1.408)	< 0.0001	1.129 (1.021–1.248)	0.018	1.111 (1.005–1.229)	0.040	1.092 (0.988–1.208)	0.086
With CKD		< 0.0001		< 0.0001		< 0.0001		0.002
< 5 mg·day‐1	1		1		1		1	
5–14 mg·day‐1	1.109 (0.883–1.394)	0.374	1.034 (0.822–1.300)	0.777	1.079 (0.858–1.357)	0.514	1.052 (0.836–1.323)	0.666
15–29 mg·day‐1	1.631 (1.293–2.056)	< 0.0001	1.485 (1.176–1.876)	< 0.0001	1.450 (1.148–1.831)	0.002	1.404 (1.111–1.774)	0.005
< 10 mg·day‐1	1		1		1		1	
10–29 mg·day‐1	1.317 (1.121–1.549)	< 0.0001	1.229 (1.045–1.447)	0.013	1.178 (1.001–1.387)	0.049	1.155 (0.982–1.349)	0.074

*Note:* Model 1: adjusted for age and sex; Model 2: adjusted for age, sex, eGFR and prior CVD; Model 3: adjusted for age, sex, eGFR, prior CVD, smoking, diabetes duration, HbA_1c_, BMI, dyslipidemia, hypertension, and use of RAS blockers.

Abbreviations: AER = albumin excretion rate, BMI = body mass index, CI = confidence interval, CKD = chronic kidney disease, CVD = cardiovascular disease, eGFR = estimated glomerular filtration rate, HbA_1c_ = haemoglobin A_1c_, HR = hazard ratio, RAS = renin‐angiotensin system.

**TABLE 4:** Cox proportional hazards regression with backward selection of variables for all‐cause mortality in all normoalbuminuric participants and in those without and with CKD, according to AER as Log2 transformed continuous variable.

	Unadjusted		Model 1		Model 2		Model 3	
	HR (95% CI)	*p*	HR (95% CI)	*p*	HR (95% CI)	*p*	HR (95% CI)	*p*
All								
AER (× 1 Log2 mg·day^−1^)	1.103 (1.066–1.141)	< 0.0001	1.059 (1.026–1.094)	< 0.0001	1.056 (1.023–1.089)	< 0.001	1.049 (1.017–1.081)	0.003
*p* AER × CKD		0.986		0.860		0.798		0.778
Without CKD								
AER (× 1 Log2 mg·day^−1^)	1.092 (1.049–1.136)	< 0.0001	1.053 (1.045–1.093)	0.007	1.049 (1.011–1.089)	0.011	1.042 (1.005–1.081)	0.027
With CKD								
AER (× 1 Log2 mg·day^−1^)	1.091 (1.026–1.160)	0.005	1.068 (1.007–1.133)	0.029	1.063 (1.004–1.127)	0.037	1.060 (1.003–1.119)	0.046

*Note:* Model 1: adjusted for age and sex; Model 2: adjusted for age, sex, eGFR and prior CVD; Model 3: adjusted for age, sex, eGFR, prior CVD, smoking, diabetes duration, HbA_1c_, BMI, dyslipidemia, hypertension, and use of RAS blockers.

Abbreviations: AER = albumin excretion rate, BMI = body mass index, CI = confidence interval, CKD = chronic kidney disease, CVD = cardiovascular disease, eGFR = estimated glomerular filtration rate, HbA_1c_ = haemoglobin A_1c_, HR = hazard ratio, RAS = renin‐angiotensin system.

## 4 Discussion

This analysis showed that, for the same level of kidney function, higher AER values within the normoalbuminuric range were associated with all‐cause mortality independent of confounding from prior CVD, CVD risk factors, and treatments. In particular, mortality risk increased linearly within the normoalbuminuric range and was significantly higher in participants with AER > 15 versus < 5 mg·day^−1^ and in those with AER 10–29 versus < 10 mg·day^−1^. These findings are consistent with previous studies showing that even AER levels below the 30 mg·day^−1^ threshold are independently associated with mortality and adverse cardiorenal outcomes in both the general population and high‐risk individuals [9, 10, 11, 12, 13].

However, while these prior studies predominantly included people with relatively preserved kidney function, virtually no data are available from studies specifically focusing on individuals with CKD. The RIACE cohort included high‐risk individuals such as those with type 2 diabetes mostly (71.2%) presenting with normoalbuminuria and with a wide range of kidney function. This allowed us to assess the impact of mildly elevated albuminuria either alone, in people without CKD, or combined with reduced eGFR, in those with the nonalbuminuric CKD phenotype. Results showed that the strength of association with mortality was higher in participants with than in those without CKD. In fact, when adjusting for multiple confounders, the risk of death increased by 40.4% and 16.7% for an AER > 15 versus < 5 mg·day^−1^ and by 1.9% and 0.8% for each 1 mg·day^−1^ increase in AER in individuals with preserved and reduced eGFR, respectively. These findings indicate that, in people with CKD, mildly elevated albuminuria may confer an increased risk of death that is additional to that attributable to reduced eGFR, thus extending the CRIC results on CKD progression in the general population to all‐cause mortality in people with type 2 diabetes.

As those from the CRIC cohort [15], our data may have important clinical implications for both prognosis and treatment of CKD. The threshold for microalbuminuria at 30 mg·day^−1^ AER (or 30 mg·g^−1^ ACR) has in fact been conventionally established based on the finding that 95% of healthy people have values below this limit, which does not necessarily mean that lower levels are ‘normal’ and that they are not associated with an increased risk of adverse outcomes and hence do not require treatment. However, despite evidence from previous studies that even albuminuria within the normoalbuminuric range is associated with adverse outcomes [9, 10, 11, 12, 13], guidelines for people with type 2 diabetes generally recommend starting albuminuria‐lowering treatments for cardio‐renal protection only in those with micro or macroalbuminuria [25, 26]. In fact, RAS blockers are not advised as preferential anti‐hypertensive agents in individuals with normoalbuminuria, whereas they are recommended in those with micro or macroalbuminuria even in the presence of normal BP levels. Likewise, individuals with AER ≥ 200 mg·day^−1^ are prioritised for use of inhibitors of sodium glucose cotransporter 2 (SGLT‐2). However, indication for these agents has been recently extended to people with AER from normal to 199 mg·day^−1^, even if with a B evidence level [27]. This was mainly based on the results of two trials, that is, (1) the Dapagliflozin Effect on Cardiovascular Events–Thrombosis in Myocardial Infarction 58 (DECLARE‐TIMI 58) Trial, which included predominantly (∼70%) normoalbuminuric participants, the great majority of whom with preserved eGFR [28]; and (2) the Study of Heart and Kidney Protection With Empagliflozin (EMPA‐KIDNEY) Trial, which included a group of individuals (∼20%) with normoalbuminuria and an eGFR ranging from 20 to 44 mL·min^−1^·173 m^−2^ [29]. Finally, use of the non‐steroidal mineralocorticoid receptor antagonist finerenone is recommended only in people with AER ≥ 30 mg·day^−1^. This is because the presence of micro or macroalbuminuria was a key inclusion criterion of the two trials conducted in people with type 2 diabetes and CKD, that is, the Finerenone in Reducing Kidney Failure and Disease Progression in Diabetic Kidney Disease (FIDELIO‐DKD) [30] and the Finerenone in Reducing CV Mortality and Morbidity in Diabetic Kidney Disease (FIGARO‐DKD) [31], which were pooled together in the FInerenone in chronic kiDney diseasE and type 2 diabetes: Combined FIDELIO‐DKD and FIGARO‐DKD Trial programme analYsis (FIDELITY) [32].

Our data and those from the CRIC cohort [15] provide strong support for the use of albuminuria‐lowering drugs in individuals with mildly elevated albuminuria, especially in those with reduced eGFR, consistent with a recent systematic review and meta‐analysis [33]. The non‐linear relationship between AER within the normoalbuminuric range and mortality may allow identification of a new AER threshold above which risk for adverse events including all‐cause death is increased and treatment is therefore required. This might be particularly important since no specific treatment has been identified yet for the nonalbuminuric CKD phenotype, which is usually excluded from randomized clinical trials [1, 2]. However, the impact of albuminuria‐lowering treatment on mortality risk could not be evaluated in the RIACE cohort since it is not contemporary and, hence, while ∼50% of participants were on RAS blockers, consistent with guideline recommendations on the use of these agents in normoalbuminuric individuals, none of them was on an SGLT‐2 inhibitor or finerenone at baseline (with only very few participants starting an SGLT‐2 inhibitor during the follow‐up). Therefore, further studies are required to demonstrate that lowering albuminuria within the normoalbuminuric range is effective in reducing mortality, CVD events, and CKD onset and progression in normoalbuminuric individuals with type 2 diabetes. Moreover, the finding of the EMPA‐KIDNEY trial that empagliflozin reduced the long‐term eGFR slope but not the primary outcome (kidney disease progression and cardiovascular death) and other kidney secondary outcomes (kidney disease progression and total eGFR slope) in the normoalbuminuric group, likely due to the small number of events [29], suggests the need for trials of long duration.

The strengths of our study include the large sample size, the completeness of baseline and follow‐up data, and the assessment of a wide range of clinical parameters, which allowed accounting for several confounders. However, there are several limitations. First, the historical, non‐contemporary RIACE cohort did not allow evaluating the impact of albuminuria‐lowering treatments on mortality. Second, the lack of information on the causes of death did not allow detection of differences in CVD versus non‐CVD mortality. Third, measurements were not centralised, though data from different laboratories/methods were standardized, as extensively discussed in previous publications [16, 17, 20, 23]. Fourth, results may have been affected by unmeasured confounders that can affect mortality. Fifth, the study findings may not be applicable to the general ambulatory population, as only part of the individuals with type 2 diabetes attend Diabetes Clinics in Italy. Finally, the observational design makes causal interpretation impossible.

In conclusion, this analysis shows that mildly elevated albuminuria is associated with all‐cause mortality independent of eGFR and other confounders in people with type 2 diabetes. Risk of death increased non‐linearly with albuminuria and was significantly higher in participants with higher AER levels within the normoalbuminuric range, with a strength of association with mortality that was higher in individuals with than in those without CKD These findings provide further support to the use of albuminuria‐lowering drugs also in people with mildly elevated albuminuria, though trials of adequate duration specifically targeting this population are required to verify whether treatment is effective in reducing adverse cardiorenal outcomes also in these individuals.

## Author Contributions

**Monia Garofolo:** conceptualization, data curation, formal analysis, investigation, resources, visualization, writing – review and editing. **Giuseppe Penno:** conceptualization, data curation, formal analysis, investigation, resources, visualization, writing – review and editing. **Anna Solini:** conceptualization, data curation, investigation, resources, writing–review and editing. **Emanuela Orsi:** conceptualization, data curation, investigation, resources, writing – review and editing. **Martina Vitale:** investigation, resources, writing – review and editing. **Veronica Resi:** investigation, resources, writing – review and editing. **Enzo Bonora:** investigation, resources, writing – review and editing. **Cecilia Fondelli:** investigation, resources, writing – review and editing. **Roberto Trevisan:** investigation, resources, writing – review and editing. **Monica Vedovato:** investigation, resources, writing – review and editing. **Antonio Nicolucci:** data curation, formal analysis, software, writing – review and editing. **Giuseppe Pugliese:** conceptualization, data curation, formal analysis, funding acquisition, project administration, investigation, resources, supervision, validation, visualization, writing – original draft. All authors gave final approval of the version to be published and agreed to be accountable for all aspects of the work in ensuring that questions related to the accuracy or integrity of any part of the work are appropriately investigated and resolved. Giuseppe Pugliese is the guarantor of this work and, as such, had full access to all of the data in the study and takes responsibility for the integrity of the data and the accuracy of the data analysis.

## Ethics Statement

The study was conducted in accordance with the Declaration of Helsinki. The research protocol was approved by the ethics committee of the coordinating centre (Sant’Andrea University Hospital, Rome, Italy, N.4306) and subsequently by the ethics committee of each participating centre. Participants provided informed consent.

## Conflicts of Interest

All authors have completed and submitted the Conflicts of Interest Disclosure form. Monia Garofolo reported consultant fees from Eli Lilly, and lecture fees from Eli Lilly, Merck Sharp & Dohme, and Novo Nordisk. Giuseppe Penno reported consultant fees from Bayer and Eli Lilly, and lecture fees from AstraZeneca, Boehringer Ingelheim, Eli‐Lilly, Merck Sharp & Dohme, Mundipharma, Novo Nordisk, and Takeda. Anna Solini reported consultant fees from Bayer, Novo Nordisk and Sankyo, and lecture fees from Bayer, Boehringer Ingelheim, Eli Lilly, Novo Nordisk, and Sanofi‐Aventis. Emanuela Orsi reported consultant fees from Eli Lilly and Novo Nordisk, and lecture fees from Astellas. Martina Vitale reported lecture fees from MundiPharma and Novo Nordisk. Veronica Resi reported lecture fees from Astra‐Zeneca, Eli Lilly, and Sanofi‐Aventis. Enzo Bonora reported consultant fees from Abbott, Bayer, Becton Dickinson, Boehringer Ingelheim, Daiichi‐Sankyo, Eli Lilly, and Novo Nordisk. Cecilia Fondelli reported lecture fees from Abbot, Boehringer Ingelheim, Daiichi Sankyo, Eli Lilly, Merck Sharp & Dohme, Mundipharma, and Theras Lifetech. Roberto Trevisan reported consultant fees from AstraZeneca, Bayer, Boehringer Ingelheim, Eli Lilly, Merck Sharp & Dohme, Novo Nordisk, and Sanofi‐Aventis, and lecture fees from AstraZeneca, Boehringer Ingelheim, Eli Lilly, and Novo Nordisk. Monica Vedovato reported lecture fees from Lifescan and Novo Nordisk. Antonio Nicolucci reported consultant fees from AstraZeneca, lecture fees from Eli Lilly, Medtronic, and Novo Nordisk, and grant support from AlfaSigma, Novo Nordisk, Pikdare, Sanofi, Shionogi, SOBI, and Theras. Giuseppe Pugliese reported consultant fees from Abbot, Bayer, and Novo Nordisk, and lecture fees from AstraZeneca, Boehringer Ingelheim, Eli Lilly, Mundipharma, and Novo Nordisk. No other disclosures were reported.

## Peer Review

The peer review history for this article is available at https://www.webofscience.com/api/gateway/wos/peer-review/10.1002/dmrr.70061.

## Supporting information

Supporting Information S1

## Data Availability

The data that support the findings of this study are available on reasonable request from the corresponding author. The data are not publicly available due to privacy or ethical restrictions.
