# Supplementary material for: Association of Albuminuria Within the Normoalbuminuric Range With All‐Cause Mortality in People With Type 2 Diabetes
Source: Diabetes Metab Res Rev. 2025 Jun 25;41(5):e70061. doi: 10.1002/dmrr.70061 (PMC12188507; doi:10.1002/dmrr.70061)
Supplement: Supplementary file 1 — Supporting Information S1 [file DMRR-41-e70061-s001.docx]

**Supporting Information**

**Association of albuminuria within the normoalbuminuric range with all-cause mortality in people with type 2 diabetes**

**The Renal Insufficiency And Cardiovascular Events (RIACE) Italian Multicenter Study**

Monia Garofolo, Giuseppe Penno, Anna Solini, Emanuela Orsi, Martina Vitale, Valeria Grancini, Enzo Bonora, Cecilia Fondelli, Roberto Trevisan, Monica Vedovato, Antonio Nicolucci, and Giuseppe Pugliese, for the Renal Insufficiency And Cardiovascular Events (RIACE) Study Group.

**Corresponding Author:** Giuseppe Pugliese, University of Rome La Sapienza, Rome, Italy; [giuseppe.pugliese@uniroma1.it](mailto:giuseppe.pugliese@uniroma1.it).

**The RIACE** **Study Group.**

**Supplementary Table S1.**

**Supplementary Figure 1**

**The RIACE Study Group.** List of the RIACE Investigators.

**The RIACE Steering Committee**

Giuseppe Pugliese (Coordinator), Giuseppe Penno (Secretary), Anna Solini, Enzo Bonora, Emanuela Orsi, Roberto Trevisan, Luigi Laviola, Antonio Nicolucci.

**Participating Diabetes Centers**

- Azienda Ospedaliera Sant'Andrea, Roma (Coordinating Center): Giuseppe Pugliese, Lucilla Bollanti, Elena Alessi, Martina Vitale, Jonida Haxhi, and Lorenza Mattia.
- Ospedale Le Molinette, Torino: Paolo Cavallo-Perin, Gabriella Gruden, and Bartolomeo Lorenzati.
- Ospedale San Luigi Gonzaga, Orbassano: Franco Cavalot, Mariella Trovati, Leonardo Di Martino, and Fabio Mazzaglia.
- Ospedale San Raffaele, Milano: Giampaolo Zerbini, Valentina Martina, Silvia Maestroni, and Valentina Capuano.
- IRCCS “Cà Granda – Ospedale Maggiore Policlinico”, Milano: Emanuela Orsi, Valeria Grancini, and Veronica Resi.
- Ospedale San Paolo, Milano: Antonio Pontiroli, Annamaria Veronelli, and Barbara Zecchini.
- Ospedale San Giuseppe, Milano: Maura Arosio, Laura Montefusco, and Guido Adda.
- ASST - Ospedale Papa Giovanni XXIII, Bergamo: Roberto Trevisan, Anna Corsi, and Mascia Albizzi.
- Ospedale Maggiore, Verona: Enzo Bonora, and Giacomo Zoppini.
- Policlinico Universitario, Padova: Angelo Avogaro, and Monica Vedovato.
- Ospedale Cisanello, Azienda Ospedaliero-Universitaria Pisana, Pisa: Giuseppe Penno, Laura Pucci, Daniela Lucchesi, Eleonora Russo, and Monia Garofolo.
- Ospedale Santa Chiara, Azienda Ospedaliero-Universitaria Pisana, Pisa: Anna Solini.
- Ospedale Le Scotte, Siena: Francesco Dotta, Cecilia Fondelli, and Laura Nigi.
- Policlinico Umberto I, Roma: Susanna Morano, Tiziana Filardi, Irene Turinese, and Marco Rossetti.
- Ospedale S. Maria Goretti, Latina: Raffaella Buzzetti and Chiara Foffi.
- Ospedali Riuniti, Foggia: Mauro Cignarelli, Olga Lamacchia, Sabina Pinnelli, and Lucia Monaco.
- Policlinico Universitario, Bari: Francesco Giorgino, Luigi Laviola, and Annalisa Natalicchio.
- Policlinico Mater Domini, Catanzaro: Giorgio Sesti and Francesco Andreozzi.
- Policlinico Monserrato, Cagliari: Marco Giorgio Baroni, Giuseppina Frau, and Alessandra Boi.

**Supplementary Table S1.** Baseline clinical features in all normoalbuminuric participants and in those without and with CKD, stratified by two AER subcategories (<10 and 10-29 mg·day^-1^).

|  | **All (n. 11,460)** | | | **Without CKD (n. 9,984)** | | | **With CKD (n. 1,476)** | | |
| --- | --- | --- | --- | --- | --- | --- | --- | --- | --- |
|  | **<10** | **10*-29*** | ***p*** | **<10** | **10*-29*** | ***p*** | **<10** | **10*-29*** | ***p*** |
| **N (%)** | 5,990 (52.3) | 5,470 (47.7) |  | 5,299 (53.1) | 4,685 (46.9) |  | 691 (46.8) | 785 (53.2) |  |
| **AER, mg·day^-1^** | 5.37  (3.00-7.50) | 15.96  (12.40-20.44) |  | 5.40  (3.00-7.50) | 15.84  (12.30-20.16) |  | 5.14  (3.00-7.30) | 16.78  (12.87-21.56) |  |
| **Age, years** | 65.3±10.3 | 66.8±10.2 | <0.0001 | 64.2±10.0 | 65.5±9.9 | <0.0001 | 74.0±7.8 | 74.7±8.3 | 0.092 |
| **Sex, n (%)** |  |  | <0.0001 |  |  | <0.0001 |  |  | 0.001 |
| **Females** | 3.015 (50.3) | 2,384 (43.6) |  | 2,567 (48.4) | 1,943 (41.5) |  | 448 (64.8) | 441 (56.2) |  |
| **Males** | 2,975 (49.7) | 3,086 (56.4) |  | 2,732 (51.6) | 2,742 (58.6) |  | 243 (35.2) | 344 /43.8) |  |
| **Smoking, n (%)** |  |  | <0.0001 |  |  | <0.0001 |  |  | 0.426 |
| **Never** | 3,960 (61.6) | 3,055 (55.9) |  | 3,243 (61.2) | 2,567 (54.8) |  | 447 (64.7) | 488 (62.2) |  |
| **Former** | 1,485 (24.8) | 1,576 (28.8) |  | 1,301 (24.6) | 1,343 (28.7) |  | 184 (26.6) | 233 (29.7) |  |
| **Current** | 815 (13.6) | 839 (15.3) |  | 755 (14.2) | 775 (16.5) |  | 60 (8.7) | 64 (8.2) |  |
| **Diabetes duration, years** | 11.9±9.4 | 12.7±9.8 | <0.0001 | 11.4±9.1 | 12.0±9.4 | 0.002 | 15.8±10.8 | 16.6±10.9 | 0.157 |
| **HbA_1c_, %** | 7.35±1.35 | 7.51±1.48 | <0.0001 | 7.33±1.35 | 7.48±1.48 | <0.0001 | 7.51±1.39 | 7.65±1.49 | 0.065 |
| **(mmol·mol^-1^)** | (56.8±14.8) | (58.6±16.2) |  | (56-6±14.7) | (58.3±16.2) |  | (58.6±15.2) | (60.1±16.3) |  |
| **BMI, kg·m^-2^** | 28.7±5.1 | 28.9±5.1 | 0.038 | 28.6±5.1 | 28.9±5.1 | 0.018 | 29.2±5.2 | 29.0±5.1 | 0.526 |
| **eWC, cm** | 101.7±10.2 | 102.3±10.3 | 0.002 | 101.6±10.2 | 102.3±10.4 | 0.001 | 102.3±10.2 | 102.2±10.1 | 0.799 |
| **Triglycerides, mmol·l^-1^** | 1.27  (0.93-1.76) | 1.31  (0.96-1.85) | <0.0001 | 1.25  (0.91-1.72) | 1.29  (0.95-1.82) | <0.0001 | 1.43  (1.06-2.04) | 1.44  (1.07-2.04) | 0.925 |
| **Total cholesterol, mmol·l^-1^** | 4.78±0.96 | 4.79±0.98 | 0.095 | 4.77±0.95 | 4.79±0.98 | 0.255 | 4.83±1.02 | 4.74±1.01 | 0.019 |
| **HDL cholesterol, mmol·l^-1^** | 1.27  (1.08-1.50) | 1.26  (1.04-1.49) | 0.001 | 1.27  (1.09-1.53) | 1.27  (1.06-1.50) | 0.006 | 1.24  (1.05-1.45) | 1.20  (1.00-1.45) | 0.088 |
| **Non-HDL cholesterol, mmol·l^-1^** | 3.46±0.92 | 3.49±0.94 | 0.083 | 3.45±0.91 | 3.49±0.94 | 0.024 | 3.56±0.96 | 3.48±0.94 | 0.016 |
| **LDL cholesterol, mmol·l^-1^** | 2.79±0.83 | 2.80±0.85 | 0.133 | 2.79±0.83 | 2.81±0.84 | 0.222 | 2.80±0.86 | 2.73±0.86 | 0.019 |
| **Dyslipidaemia, n (%)** | 4,904 (81.9) | 4.527 (82.8) | 0.212 | 4,318 (81.5) | 3,872 (82.6) | 0.132 | 586 (84.8) | 655 (83.4) | 0.474 |
| **Systolic BP, mmHg** | 136.2±17.5 | 138.3±17.6 | <0.0001 | 135.9±17.2 | 138.1±17.3 | <0.0001 | 137.8±18.8 | 139.3±18.7 | 0.107 |
| **Diastolic BP, mmHg** | 78.1±9.3 | 79.2±9.1 | <0.0001 | 78.3±9.2 | 79.5±9.1 | <0.0001 | 80.0±9.9 | 77.8±9.6 | 0.097 |
| **Mean BP, mmHg** | 97.5±10.3 | 98.9±10.3 | <0.0001 | 97.7±10.3 | 99.0±10.2 | <0.0001 | 97.2±10.7 | 98.3±10.9 | 0.055 |
| **Pulse pressure, mmHg** | 58.0±15.4 | 59.1±15.3 | <0.0001 | 57.7±15.1 | 58.6±15.0 | 0.001 | 60.8±17.5 | 61.5±16.2 | 0.402 |
| **Hypertension, n (%)** | 4,738 (79.1) | 4,539 (83.0) | <0.0001 | 4,094 (77.3) | 3,812 (81.4) | <0.0001 | 644 (93.2) | 727 (92.6) | 0.662 |
| **Anti-hyperglycemic Tx, n (%)** |  |  | <0.0001 |  |  | <0.0001 |  |  | <0.0001 |
| **Lifestyle only** | 1,044 (17.4) | 694 (12.7) |  | 944 (17.8) | 631 (13.5) |  | 100 (14.5) | 63 (8.0) |  |
| **Non-insulin** | 3,738 (62.4) | 3,507 (64.1) |  | 3,350 (63.2) | 3,051 (65.1) |  | 388 (56.2) | 456 (68.1) |  |
| **Insulin** | 1,208 (20.2) | 1.269 (23.2) |  | 1,005 (19.0) | 1,003 (21.4) |  | 203 (29.4) | 266 (33.9) |  |
| **Lipid-lowering Tx, n (%)** | 2,707 (45.2) | 20.2) | 0.844 | 2,327 (43.9) | 2,055 (43.9) | 0.959 | 380 (55.0) | 427 (54.4) | 0.818 |
| **Anti-hypertensive Tx, n (%)** | 3,859 (64.4) | 3,759 (68.7) | <0.0001 | 3,264 (61.6) | 3,084 (65.8) | <0.0001 | 595 (86.1) | 675 (86.0) | 0.947 |
| **RAS blockers, n (%)** | 3,076 (51.4) | 3,062 (56.0) | <0.0001 | 2,605 (49.2) | 2,505 (53.5) | <0.0001 | 471 (68.2) | 557 (71.0) | 0.244 |
| **Anti-platelet Tx, n (%)** | 2.068 (34.5) | 2,142 (39.2) | <0.0001 | 1,705 (32.2) | 1,723 (36.8) | <0.0001 | 363 (52.5) | 419 (53.4) | 0.746 |
| **Anti-coagulant Tx, n (%)** | 161 (2.7) | 228 (4.2) | <0.0001 | 114 (2.2) | 150 (3.2) | 0.001 | 47 (6.8) | 78 (9.9) | 0.031 |
| **Serum creatinine, µmol/l** | 79.1±20.2 | 81.8±28.7 | <0.0001 | 74.8±14.5 | 75.6±21.0 | 0.017 | 112.6±25.5 | 118.6±38.7 | <0.0001 |
| **eGFR, ml·min^-1^·1.73m^-2^** | 83.2±18.3 | 82.1±19.6 | 0.001 | 87.7±14.0 | 87.8±14.2 | 0.658 | 49.1±8.7 | 47.9±10.1 | 0.017 |
| **DR, n (%)** |  |  | <0.0001 |  |  | <0.0001 |  |  | 0.436 |
| **No** | 5,005 (83.6) | 4,397 (80.4) |  | 4,466 (84.3) | 3.807 (81.3) |  | 539 (78.0) | 590 (75.2) |  |
| **Non-advanced** | 597 (10.0) | 662 (12.1) |  | 512 (9.7) | 553 (11.8) |  | 85 (12.3) | 110 (14.0) |  |
| **Advanced** | 388 (6.5) | 410 (7.5) |  | 321 (6.1) | 325 (6.9) |  | 67 (9.7) | 85 (10.8) |  |
| **CVD, n (%)** |  |  |  |  |  |  |  |  |  |
| **Any** | 1,069 (17.8) | 1,196 (21.9) | <0.0001 | 846 (16.0) | 924 (19.7) | <0.0001 | 223 (32.3) | 272 (34.6) | 0.334 |
| **Acute myocardial infarction** | 557 (9.3) | 581 (10.6) | 0.018 | 438 (8.3%) | 442 (9.4) | 0.040 | 118 (17.2) | 139 (17.7) | 0.806 |
| **Coronary revascularization** | 490 (8.2) | 549 (10.0) | 0.001 | 381 (7.2) | 428 (9.1) | <0.0001 | 109 (15.8) | 121 (15.4) | 0.849 |
| **Any coronary event** | 748 (12.5) | 821 (15.0) | <0.0001 | 589 (11.1) | 629 (13.4) | <0.0001 | 159 (23.0) | 192 (24.5) | 0.514 |
| **Stroke** | 141 (2.4) | 160 (2.9) | 0.056 | 109 (2.1) | 122 (2.6%) | 0.070 | 32 (4.6) | 38 (4.8) | 0.850 |
| **Carotid revascularization** | 258 (4.3) | 241 (4.4) | 0.796 | 197 (3.7) | 173 (3.7) | 0.947 | 61 (8.8) | 68 (8.7) | 0.911 |
| **Any cerebrovascular event** | 378 (6.3) | 382 (7.0) | 0.148 | 291 (5.5) | 282 (6.0) | 0.258 | 87 (12.6) | 100 (12.7) | 0.932 |
| **Ulcer/gangrene/ amputation** | 104 (1.7) | 147 (2.7) | 0.001 | 77 (1.5) | 107 (2.3) | 0.002 | 27 (3.9) | 40 (5.1) | 0.274 |
| **Lower limb revascularization** | 123 (2.1) | 123 (2.2) | 0.471 | 91 (1.7) | 87 (1.9) | 0.599 | 32 (4.6) | 36 (4.6) | 0.967 |
| **Any peripheral event** | 215 (3.6) | 254 (4.6) | 0.004 | 159 (3.0) | 187 (4.0) | 0.007 | 56 (8.1) | 67 (8.5) | 0.765 |
| **Comorbidities, n (%)** |  |  |  |  |  |  |  |  |  |
| **Any** | 954 (15.9) | 955 (17.5) | 0.028 | 834 (15.7) | 776 (16.6) | 0.263 | 120 (17.4) | 179 (22.8) | 0.010 |
| **COPD** | 194 (3.2) | 234 (4.3) | 0.003 | 155 (2.9) | 176 (3.8) | 0.021 | 39 (5.6) | 58 (7.4) | 0.177 |
| **Chronic liver disease** | 496 (8.3) | 452 (8.3) | 0.973 | 442 (8.3) | 383 (8.2) | 0.763 | 54 (7.8) | 69 (8.8) | 0.499 |
| **Cancer** | 348 (5.8) | 352 (6.6) | 0.073 | 307 (5.8) | 291 (6.2) | 0.380 | 41 (5.9) | 71 (9.0) | 0.024 |

Data are expressed as mean±SD or median (interquartile range), for continuous variables, and number of cases (percentage), for categorical variables. CKD = chronic kidney disease; AER = albumin excretion rate; HbA_1c_ = hemoglobin A_1c_; BMI = body mass index; eWC = estimated waist circumference; BP = blood pressure; Tx = treatment; eGFR = estimated glomerular filtration rate; DR = diabetic retinopathy; CVD = cardiovascular disease; COPD = chronic obstructive pulmonary disease.

**
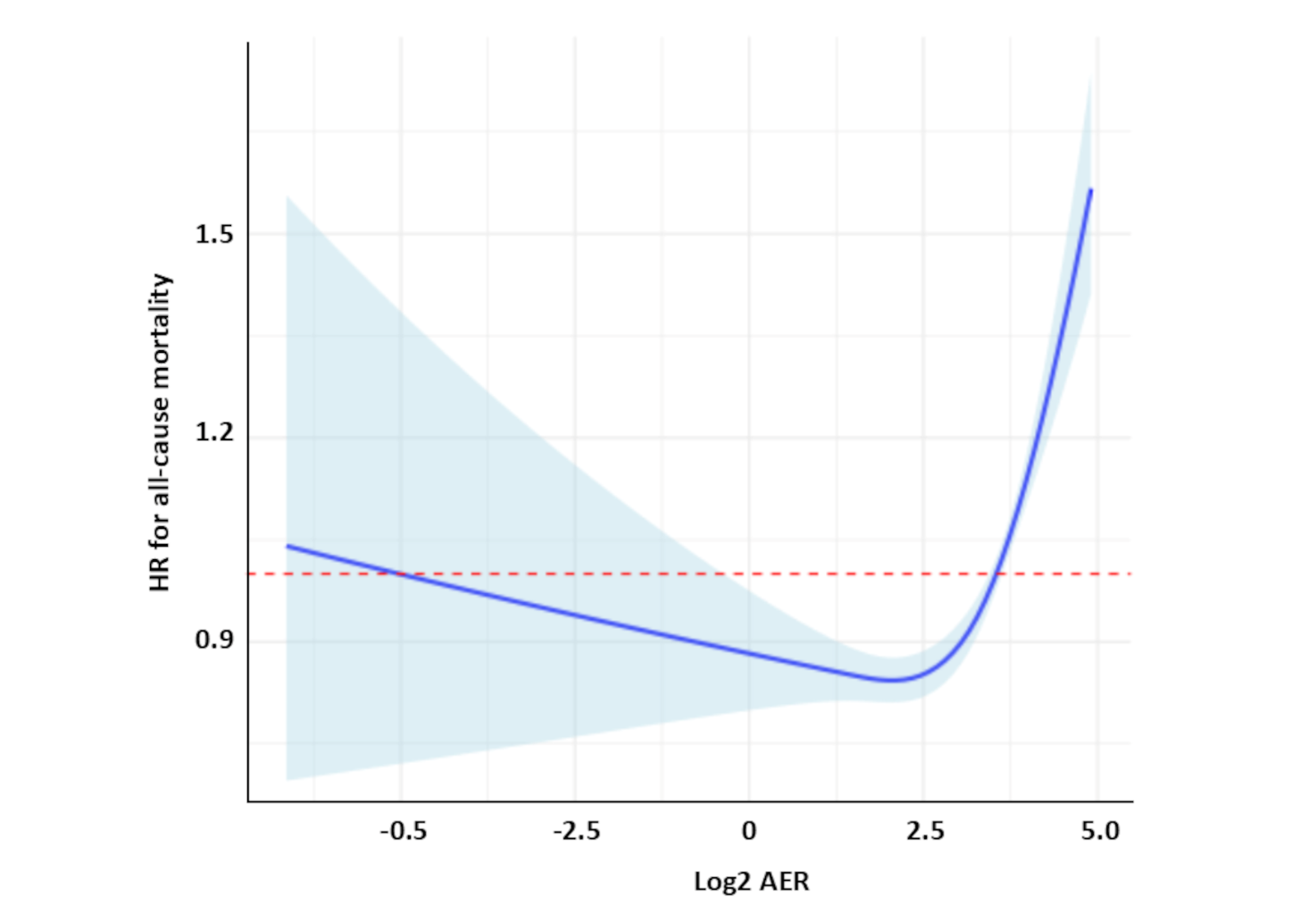
**

**Supplementary Figure 1.** Association between Log2 AER and all-cause mortality by Cox proportional hazards regression with restricted cubic splines. The blue line represents the estimated HR, the shaded area indicates the 95% CI, and the red dashed line at HR = 1 marks the point of no association. AER = albumin excretion rate; HR = hazard ratio; CI = confidence interval.
